# Deep Learning Transformer Models for Building a Comprehensive and Real-time Trauma Observatory: Development and Validation Study

**DOI:** 10.2196/40843

**Published:** 2023-01-12

**Authors:** Gabrielle Chenais, Cédric Gil-Jardiné, Hélène Touchais, Marta Avalos Fernandez, Benjamin Contrand, Eric Tellier, Xavier Combes, Loick Bourdois, Philippe Revel, Emmanuel Lagarde

**Affiliations:** 1 Unit 1219 Bordeaux Public Health Center Institut National de la Santé et de la Recherche Médicale Bordeaux France; 2 Emergency Department Bordeaux University Hospital Bordeaux France; 3 Statistics in Systems Biology and Translational Medicine Team University of Bordeaux Institut National de Recherche en Sciences et Technologies du Numérique Talence France

**Keywords:** deep learning, public health, trauma, emergencies, natural language processing, transformers

## Abstract

**Background:**

Public health surveillance relies on the collection of data, often in near-real time. Recent advances in natural language processing make it possible to envisage an automated system for extracting information from electronic health records.

**Objective:**

To study the feasibility of setting up a national trauma observatory in France, we compared the performance of several automatic language processing methods in a multiclass classification task of unstructured clinical notes.

**Methods:**

A total of 69,110 free-text clinical notes related to visits to the emergency departments of the University Hospital of Bordeaux, France, between 2012 and 2019 were manually annotated. Among these clinical notes, 32.5% (22,481/69,110) were traumas. We trained 4 transformer models (deep learning models that encompass attention mechanism) and compared them with the term frequency–inverse document frequency associated with the support vector machine method.

**Results:**

The transformer models consistently performed better than the term frequency–inverse document frequency and a support vector machine. Among the transformers, the GPTanam model pretrained with a French corpus with an additional autosupervised learning step on 306,368 unlabeled clinical notes showed the best performance with a micro *F*_1_-score of 0.969.

**Conclusions:**

The transformers proved efficient at the multiclass classification of narrative and medical data. Further steps for improvement should focus on the expansion of abbreviations and multioutput multiclass classification.

## Introduction

### Background

The objective of public health surveillance is to describe a health event in the population to estimate its burden based on its characteristics (incidence, prevalence, survival, and mortality) and evolution. This surveillance contributes to the definition, implementation, monitoring, and evaluation of public health policies. It must also be able to alert to the emergence of new threats to public health (infectious or environmental in origin and natural or terrorist) and monitor and evaluate the impact of known and expected events (seasonal epidemics) or unexpected events (industrial disasters and extreme weather events) on the health of the population. Public health surveillance relies on the collection of data, often in near real time.

The SurSaUD (Surveillance Sanitaire des Urgences et des Décès) syndromic surveillance system was created for the purpose of public health surveillance in France in 2004 by Santé Publique France, the French National Public Health Agency. The SurSaUD system collects daily data from 4 sources: emergency departments (EDs; OSCOUR ED network) [1], emergency general practitioners (SOS Médecins network), crude mortality (civil status data), and electronic death certification including causes of death [2]. Since its inception, the OSCOUR network has recorded >130 million ED visits. Data are collected by the direct extraction of information from patients’ electronic health records (EHRs) in a common format for the entire territory and transmitted to Santé Publique France via the OSCOUR network. Owing to the coding of the main diagnosis (International Classification of Diseases [ICD] 10th Revision codes) and progressive improvement of data quality [3], the network can establish real-time monitoring of public health events such as epidemics of influenza, gastroenteritis, or bronchiolitis [4-7]. This is one of the tools currently used to monitor responses to the COVID-19 epidemic in France.

Approximately one-third of ED visits in France are the result of trauma [8]. Trauma is a major cause of mortality and morbidity worldwide [7]. In 2017, trauma and injury accounted for 7.01% (range 6.75%-7.33%) of the deaths in France [9]. Unfortunately, little information is available regarding trauma; although we can know the nature of the main injury, nothing is known about the mechanism (road accident, assault, suicide, etc). However, this information is available in the EHR but in a free-text form. In fact, each time a patient visits the ED, the nurse in charge of reception and orientation and the physician in charge of the first consultation enter a text called clinical note, which describes the reasons for the patient’s visit and the circumstances in which the symptoms occurred. To add the trauma mechanism to the data collected by the OSCOUR network, a manual classification by health professionals would be time consuming and require multiple resources. Given the nature of the data (free text, unstructured, and containing abbreviations) to be processed and the objective (classification), artificial intelligence with deep learning, particularly automatic language processing, seems to be indicated.

Natural language analysis has seen a recent breakthrough with the introduction of deep learning, in particular, the transformer architecture. Introduced in 2017 by Google and proposed in the article “Attention is All You Need” by Vaswani et al [10], transformers have an architecture that allows the implementation of a mechanism for processing the sequence of tokens (a token is an instance of a sequence of characters in a particular document that are grouped together as a semantic unit useful for language processing) that form a sentence in a self-attentive manner, that is, relating each of these tokens to each of the others in the sentence. They have the particularity of being able to be pretrained on a corpus of text, which can be very large because it does not require a coding stage. This phase leads to a generative model that is capable, for example, of constructing artificial text by iteration. The Bidirectional Encoder Representations from Transformers (BERT) are one of these transformer-type models pretrained on large corpora of text [11]. The BERT model is a bidirectional transformer composed of only encoder blocks. The particularity of BERT model is that it learns information from both the right and left sides of a token’s context during the pretraining and training phases. BERT is composed of a stack of 12 identical layers. Each layer consists of 2 sublayers. The first is a multihead self-attention mechanism, and the second is a simple, position-wise fully connected feed-forward network. In other words, the text encoder converts text into a numeric representation. On many tasks, including text classification, its performance is systematically superior to that of the convolutional and autoregressive models used until then [11].

French derivatives of the BERT model such as FlauBERT [12] and CamemBERT [13] have been trained on very large and diverse French corpora. FlauBERT is a French BERT trained on a very large and heterogeneous French corpus. Models of different sizes were trained using the Jean Zay supercomputer of the Centre National de la Recherche Scientifique; there are 3 sizes: small (54 million parameters), base cased (138 million parameters) and uncased (137 million parameters), as well as large (373 million parameters). CamemBERT is based on RoBERTa [14], which is an evolution of BERT in several aspects, including the use of the masked language model as the sole pretraining objective. Similar to FlauBERT, CamemBERT is available in different sizes: base (110 million parameters) and large (335 million parameters); moreover, it can be trained on different training corpora such as OSCAR (either 138 GB or 4 GB of text) [15], CCNET (either 135 GB or 4 GB) [16], or French Wikipedia (4 GB).

One of the most interesting examples of transformer architecture is Generative Pretrained Transformer-2 (GPT-2), released by OpenAI in 2019. GPT-2 is a large transformer-based model composed solely of decoder blocks, with 1.5 billion parameters on its extra-large version, and trained on a data set of 8 million web pages to predict the next word from the previous words [17]. A total of 3 other sizes of GPT-2 were released before the largest: 124 (small), 355 (medium), and 774 (large) million parameters. This model’s ability to generate text attracted the attention of the community quickly because of the difficulty in distinguishing the produced artificial texts from the texts written by humans, suggesting that some of the meaning present in natural language was embedded. Moreover, beyond its ability to generate coherent texts, GPT-2 can perform other tasks such as answering questions or classifying documents. As with BERT, the conservation of several self-attention block weights from a pretrained model is sufficient to transfer contextual representations into another data set. The training of the GPT-2 model is thus carried out in 2 distinct phases. The first phase of self-supervised generative pretraining consists of the reading of a corpus of texts. This leads to the ability to generate texts automatically. The second supervised training phase consists of resuming the learning process in a corpus of annotated texts to create a system capable of performing specific tasks (eg, classification). BelGPT2 is a Belgian small GPT-2 pretrained on a French corpus of 60 GB (Common Crawl, Project Gutenberg, Wikipedia, EuroPARL, etc) that was released at the end of 2020 [18].

### Related Work

Extracting mechanisms and types of traumas are a matter of multiclass classification. Multiclass classification of French medical data involves a wide variety of techniques. For example, for the 2018 Conference and Labs of the Evaluation Forum eHealth task 1 challenge [19], the objective of which was to extract ICD 10th Revision codes from the death certificates provided by the Centre for Epidemiology of Medical Causes of Death, Cossin et al [20] tested an approach based on ontologies, whereas Flicoteaux et al [21] proposed an approach using a probabilistic convolutional neural network (CNN), and Ive et al [22] resorted to the association of a recurrent neural network with a CNN. By contrast, Metzger et al classified free-text clinical notes from ED related to suicide attempts using random forest and naive Bayes–type algorithms [23]. Recent studies have shown the effectiveness of transformers in classification tasks for EHR free-text data such as ICD coding [24,25], phenotyping [26], and readmission prediction [27]. Therefore, within the framework of the TARPON (Traitement Automatique des Résumés de Passage aux urgences dans le but de créer un Observatoire National) project, which aims to demonstrate the feasibility of setting up a national observatory of trauma, we propose here to compare the performances of several transformer models in the classification of ED visits for trauma based on clinical notes from the adult ED of the Bordeaux University Hospital. We compared the transformers FlauBERT, CamemBERT, BelGPT2, and a French GPT-2 model pretrained on a domain-specific corpus called GPTanam with term frequency–inverse document frequency (TF-IDF)/support vector machine (SVM), which was used as a baseline model. To the best of our knowledge, no previous performance evaluation of multiple transformers’ classification application has been conducted on complex and unstructured clinical data from ED combining common French language, medical data, and jargon.

## Methods

### Medical Ethics Regulations and General Data Protection Regulation

This study was authorized by the Bordeaux University Hospital Ethical Board under number GP-CE2021-21. A data management plan was created and reviewed by the privacy security board to meet the institutional and national requirements in France for General Data Protection Regulation compliance.

### Database

Clinical notes were extracted from the EHR of the adult ED stored in the information system of the University Hospital of Bordeaux, France. They correspond to 375,478 medical records of visits to the adult ED of Bordeaux Hospital from 2012 to 2020. The variables available were age, sex, date and time of the visit, the clinical note generated by the physicians or interns, and the clinical note written by the triage nurses.

### Labeling Strategy

In total, 69,110 clinical notes were randomly extracted for manual annotation. Our coding team consisted of trauma epidemiologists, emergency physicians, emergency nurses, research assistants, and biostatisticians, amounting to a total of 16 coders. The annotation phase lasted 5 months. For each clinical note, a code describing the content of the text was assigned. The annotation grid used for coding was developed for the needs of the project. The code associated with each clinical note consisted of 9 fields. The fields were as follows: “First visit (to the emergency department for this reason),” “Location (of the trauma),” “Activity (performed during the trauma),” “Type of Sport (practiced during the trauma),” “Subject under the influence,” “Notion of pre-traumatic discomfort,” “MVA (Motor Vehicle Accident)-Secondary Prevention Elements,” “MVA-Antagonist,” and “Type of trauma or Mode of travel for the MVA.” As the objective was to classify the types of trauma, we mainly used the data of the field “Type of trauma or Mode of movement for the MVA.” As the distribution of the fields was unbalanced, we created a composite variable containing 8 mutually exclusive classes to have a larger number of clinical notes per class. Therefore, we grouped certain types of traumas (ie, “Fall,” which included “Fall from own height,” “Fall from a given height,” and “Fall on stairs”). The composite variable included the following classes or labels: “Accident of exposure to body fluids (blood exposure accident, unprotected sex at risk),” “Assault,” “Motor Vehicle Accident (MVA),” “Foreign body in eyes,” “Fall (except sports),” “Sports accident,” “Intentional Injury,” and “Other trauma” as shown in Multimedia Appendix 1. The interannotator agreement was assessed with a random sample of 1000 clinical notes labeled by 2 annotators, leading to a Cohen κ score [28] of 0.84.

A sensitivity analysis was performed to study the impact of potentially ambiguous content on classification. Therefore, the test sample was reread by an expert. Potentially ambiguous content in terms of classification is defined here as the accumulation of several mechanisms or types of traumas or a major difficulty in assigning a label to a clinical note given its text.

### Corpus Statistics

In total, 22,481 manually labeled clinical notes from the Bordeaux University Hospital were included in the study. One-third (22,481/69,110) of the total annotated clinical notes were labeled as visit to the ED resulting from a trauma. The average number of sentences in the corpus was 3.25 (SD 2.56; range 1-63). The average length of clinical notes was 58 (SD 38) words, with a minimum of 1 word (eg, “Accident d’exposition au sang”) and a maximum of 630 words. The number of unique unigrams, bigrams, and trigrams were 70,99, 395,827, and 777,459, respectively.

### Models and Experiment Settings

The models selected for comparison and freely available as open-source content were a traditional machine learning model (baseline model) with TF-IDF/SVM couple as well as 3 transformer-type models pretrained on French corpora: CamemBERT [13], FlauBERT [12], and BelGPT2 [18]. We then chose the best performing model and applied a supplementary step of self-supervised training with the remaining 306,368 unlabeled clinical notes. This model is called here as GPTanam. Table 1 lists the size and configuration of each transformer model.

For TF-IDF, tokenization was performed using the National Language Toolkit package (version 3.6.6; NLTK) [29], and linear support vector classifier was applied using scikit-learn (version 0.24.1) [30]. The most frequent words (eg, “that,” “he,” and “the”) were removed. Tokenization was performed using SentencePiece [31] for CamemBERT, Byte-Pair Encoding for FlauBERT, and a byte-level Byte-Pair Encoding for both GPT-2 models [32]. The data were cleaned using regular expressions with the re package in Python (version 3.7). Unicode normalization was performed in the 8-bit Universal Character Set Transformation Format. The linear support vector classifier parameters were as follows: tolerance=1 × 10^–5^, penalty=l2, loss=squared hinge, dual optimization=true, C=1.0, multiclass strategy=one versus rest, verbose=0, and a maximum of 1000 iterations. For all 3 transformers, the optimizer was AdamW, with an epsilon of 1 × 10^–8^, and the maximum length was 512. GPTanam had training and evaluation batch sizes of 5 and a learning rate of 2 × 10^–5^. For FlauBERT and CamemBERT, the batch size was 16 for training and 20 for evaluation, and the learning rate was 5 × 10^–5^. The models were trained using the Hugging Face library under the Pytorch framework on our workstation with a single Titan RTX (Nvidia) graphics processing unit with 24 GB of video RAM. Performance analysis was done using scikit-learn and imbalance-learn (version 0.9.1).

**Table 1:** Transformer models’ sizes and configurations.

Model	Layers	Attention heads	Embedding dimension	Parameters (millions)	Pretraining corpus size (GB)
CamemBERT-base-CCNET^a^	12	12	768	110	135
FlauBERT-base-cased	12	12	768	138	71
BelGPT2	12	12	768	117	57.9
GPTanam	12	12	768	117	58.6

^a^CCNET: criss-cross attention for semantic segmentation.

### Self-supervised Learning and Fine-tuning Phase

Considering the GPTanam model, the first step comprising self-supervised learning was performed with 306,368 clinical notes with 1 epoch [33]. For all the models, a random sample of 80.80% (18,166/22,481) of the clinical notes labeled as trauma was dedicated to supervised learning. This data set was divided into a training sample (14,532/18,166, 79.99%) and a validation sample (3634/18,166, 20%) in an 80/20 ratio. We trained each model 9 times with different seeds on 7 epochs for CamemBERT and FlauBERT and 5 epochs for BelGPT2 and GPTanam. To obtain a single prediction for the 9 different executions of the chosen epoch (based on the maximum validation micro *F*_1_-score) for each model, a vote was taken.

### Test Phase

The test sample contained 19.19% (4315/22,481 records) of the labeled data set. The second reading of these clinical notes resulted in 10.82% (467/4315) being tagged as clinical notes with potentially complex or ambiguous content in terms of classification. Therefore, the analysis included both the complete test data set (4315/22,481, 20%) and the data set without complex and ambiguous content (3848/22,481, 17.11%). To obtain the probabilities for each prediction, a softmax activation layer was applied to the 4 transformer models.

### Data Sets

The label distribution among the corpus and each training, validation, and test data set are presented in Table 2. The most common type of trauma was the class “Fall” followed by “Other trauma” and “Motor Vehicle Accident.” An example of clinical notes translated from French is shown in Multimedia Appendix 2.

The median age at the time of visit was 37 (IQR 24-58—first and third quartiles) years, and 58.46% (13,143/22,481) of the patients were male. EHRs were introduced in 2012 at the Bordeaux University Hospital, which explains the lower proportion of data for this particular year. In 2019, there was a decrease in ED venues, whereas in 2020, there was a significant increase in ED venues. Table 3 summarizes the characteristics of the train, validation, and test data sets for the study population. The distribution of the variables age, sex, and year of venues at the ED were comparable among the 3 data sets.

**Table 2:** Label distribution among train, validation, and test data sets.

Type of trauma	Train data set (n=14,532, 64.64%), n (%)	Validation data set (n=3634, 16.16%), n (%)	Test data set (n=4315, 19.19%), n (%)	Total (N=22,481, 100%), n (%)
Accident of exposure to bodily fluids	132 (0.91)	40 (1.1)	41 (1)	213 (0.9)
Assault	1587 (10.92)	393 (10.81)	498 (11.54)	2478 (11.02)
MVA^a^	2028 (13.95)	495 (13.62)	568 (13.16)	3091 (13.75)
Foreign body in eye	642 (4.42)	180 (5)	186 (4.31)	1008 (4.48)
Fall	4778 (32.87)	1162 (31.97)	1554 (36.01)	7494 (33.33)
Sport accident	1311 (9)	341 (9.38)	371 (8.59)	2023 (9)
Intentional injury	341 (2.34)	73 (2)	112 (2.59)	526 (2.33)
Other trauma	3713 (25.55)	950 (26.14)	985 (22.82)	5648 (25.12)

^a^MVA: motor vehicle accident.

**Table 3:** Train, validation, and test data set characteristics.

			Train data set (n=14,532)		Validation data set (n=3634)	Test data set (n=4315)	Total (N=22,481)
Age (years), median (IQR^a^)			37 (24-58)		37 (24-57)	37 (24-58)	37 (24-58)
Sex (male), n (%)			8486 (58.39)		2181 (60.01)	2476 (57.38)	13,143 (58.46)
**Year of ED^b^ venue, n (%)**							
	2012	218 (1.5)		52 (1.43)		66 (1.52)	336 (1.49)
	2013	1389 (12.2)		359 (12.4)		418 (12.3)	2166 (12.2)
	2014	1444 (12.6)		385 (13.3)		386 (11.3)	2215 (12.3)
	2015	1502 (13.1)		326 (11.2)		425 (12.5)	2253 (12.6)
	2016	1419 (12.4)		365 (12.6)		426 (12.6)	2210 (12.3)
	2017	1493 (13.1)		370 (12.8)		461 (13.5)	2324 (12.9)
	2018	1425 (12.5)		405 (13.9)		474 (13.9)	2304 (13.5)
	2019	690 (6)		175 (6)		218 (6.4)	1083 (6.2)
	2020	1856 (16.2)		468 (16.1)		532 (15.6)	2856 (16)
	Missing values	3118 (27.3)		737 (25.4)		899 (26.4)	4724 (20.9)

^a^IQR: first and fourth quartiles are given.

^b^ED: emergency department.

### Performance Criteria

The measures chosen were macro-average precision and micro *F*_1_-score, which, in the multiclass framework, are equal to accuracy. For the following equations, *n* is the number of samples (clinical notes), TP is true positive, FP is false positive, and FN is false negative.

#### Macro-Average Precision

Precision expresses the proportion of units a model classifies as positive that are actually positive. In other words, precision indicates how much one can trust the model when it predicts that a record is classified in a given class. In the case of multiclass classification, the macro-average precision over all *i* classes can be evaluated by macro-averaging, wherein the precision over each *i* class is first calculated and then the precisions over all *n* classes are averaged. There is no relation to class size, as classes of different sizes are also weighted in the numerator. This implies that the effect of larger classes is as important as that of smaller ones. Therefore, each clinical note is equally important with this measure [34].



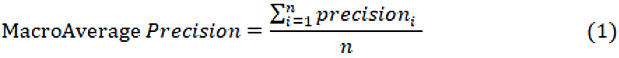



#### Micro F1-Score

*F*_1_-score is defined as the harmonic mean of precision and recall in binary class problem. To extend the *F*_1_ measure to multiclasses, 2 types of average, microaverage and macro-average, are commonly used. In microaveraging, the *F*_1_ measure is computed globally over all class decisions, with precision and recall being obtained by summing over all individual decisions. The microaveraged *F*_1_ measure gives equal weight to each clinical note and is, therefore, considered as an average over all the clinical note or category pairs [35].



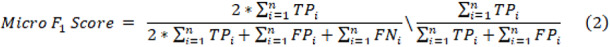



### Data Security

Identifying information was found in the data set. Therefore, we deidentified all clinical notes using named entity recognition with FlauBERT. Data processing and computing were conducted within the facilities of the ED of the University Hospital of Bordeaux, which have received regulatory clearance to host and exploit databases with personal and medical data. All the patients from whom information was retrieved were aged ≥15 years.

### Error Analysis

An error analysis was performed with unigrams and bigrams for the best performing model. All misclassified clinical notes were read by an expert to determine whether the human annotation labels were appropriate.

## Results

### Fine-tuning the Performance of Models

**Figure 1:**
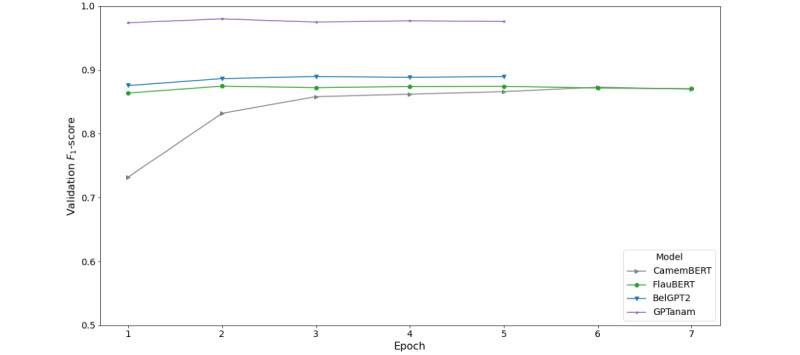
F1-score curves for CamemBERT, FlauBERT, BelGPT2 and GPTanam on the validation dataset.

Unlike statistical methods such as TF-IDF, the supervised fine-tuning of transformer models is time consuming and can be greatly accelerated by the use of graphics processing units. The self-supervised fine-tuning step for the GPTanam model required approximately 12 hours. At that point, GPTanam could generate artificial clinical notes, as seen in Multimedia Appendix 3, that could not be easily differentiated from the original ones. One epoch of supervised fine-tuning required 15, 16, 19, and 18 minutes for CamemBERT, FlauBERT, BelGPT2, and GPTanam, respectively. When looking deeper into each transformer model’s *F*_1_-scores on the validation data set, Figure 1 shows that CamemBERT reached its maximum *F*_1_-score (0.873) at epoch 6, FlauBERT achieved an *F*_1_-score of 0.874 at epoch 5, BelGPT2 reached its peak (0.890) faster at epoch 3, and GPTanam reached 0.980 at epoch 2. Moreover, GPTanam’s *F*_1_-score on the validation data set was the highest among the 4 transformer models. We conjecture that a self-supervised step on a domain-specific corpus for GPTanam contributed to the learning of the semantic representations, which resulted in a faster convergence in the learning of the classification task.

### Performance of Models

The average macro precision and micro *F*_1_-scores were systematically higher for the transformers than for the TF-IDF/SVM couple on the complete test data set, as shown in Table 4. Among the transformers, GPTanam achieved an average micro *F*_1_-score of 0.969, outperforming CamemBERT, FlauBERT, and BelGPT2, for which average *F*_1_-scores were 0.878, 0.873, and 0.887, respectively. The macro-average precision was higher than the *F*_1_-score in almost all cases, except for TF-IDF/SVM, for which the macro precision was lower than the micro *F*_1_-score (macro precision=0.860 and micro *F*_1_-score=0.864).

The distribution of *n* clinical notes per class was not balanced, and the micro *F*_1_ scores were, in all cases, lower in the classes where *n* was lower. Concerning the micro *F*_1_-score of the different classes, GPTanam had higher scores than the other transformers and TF-IDF. The performance of GPTanam was high for all classes except for intentional injuries; we assumed that this might be associated with the semantic heterogeneity and variety of the class. Indeed, this class encompassed self-harm (self-mutilation, punching due to rage, and self-stabbing) and suicide attempts (shooting, alcohol or drug poisoning, and car crashing), with few examples per injury. By contrast, classes such as motor vehicle accident (MVA) and fall have semantic consistency with a larger number of examples. The confusion matrix is shown in Multimedia Appendix 4. An error analysis of the intentional injury class, as well as the other classes, is provided in the next section.

**Table 4:** Micro F1-scores for all classes and models with microaverage F1-scores and macro-average precision on the complete test data set.

Type of trauma	Test data set (n=4315), n (%)	Micro *F*_1_-scores				
		TF-IDF^a^/SVM^b^	CamemBERT	FlauBERT	BelGPT2	GPTanam
Accident of exposure to bodily fluids	41 (1)	0.83	0.84	0.84	0.83	*0.91* ^c^
Assault	498 (11.54)	0.9	0.91	0.92	0.91	*0.96*
MVA^d^	568 (13.16)	0.91	0.90	0.91	0.91	*0.97*
Foreign body in eye	186 (4.3)	0.79	0.84	0.82	0.82	*0.97*
Fall	1554 (36.01)	0.9	0.92	0.91	0.92	*0.98*
Sport accident	371 (8.6)	0.82	0.83	0.83	0.85	*0.94*
Intentional injury	112 (2.6)	0.75	0.76	0.73	0.77	*0.84*
Other trauma	985 (22.8)	0.8	0.83	0.82	0.85	*0.98*
Micro *F*_1_-score	N/A^e^	0.864	0.878	0.873	0.887	*0.969*
Macro precision	N/A	0.860	0.880	0.880	0.89	*0.970*

^a^TF-IDF: term frequency–inverse document frequency.

^b^SVM: support vector machine.

^c^The best *F*_1_-scores are in italic.

^d^MVA: motor vehicle accident.

^e^N/A: not applicable.

### Error Analysis

The error analysis results are presented in Textbox 1.

Removing complex and ambiguous clinical notes were associated with an increase of performance for all the models; the average gain of F1-scores was 0.04 for TF-IDF/SVM, CamemBERT, FlauBERT, and BelGPT2. The average gain of the micro F1-score was 0.01 for GPTanam, which seems to be more robust in classifying complex and ambiguous content.

**Figure 2:**
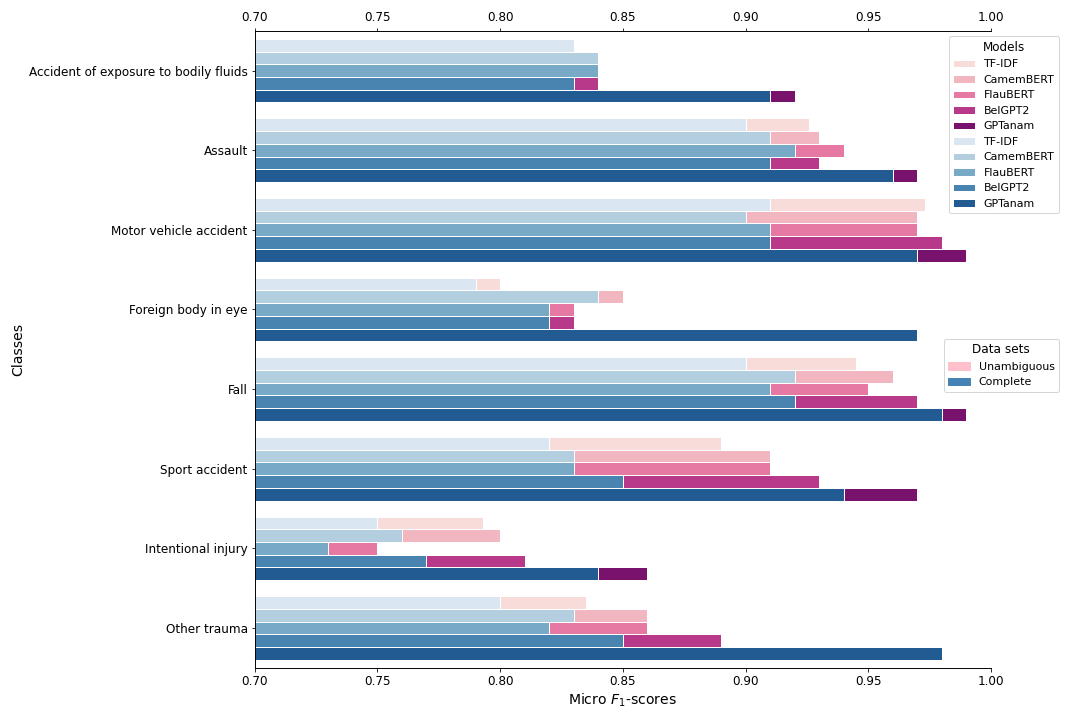
Plot of micro F1-scores of all models for each class for both the complete test data set (blue bars) and the test data set without potentially ambiguous content as regard to its classification (pink bars). TF-IDF: term frequency–inverse document frequency.

The difference in performance when potentially complex and ambiguous content was considered was greater for TF-IDF/SVM, CamemBERT, FlauBERT, and BelGPT2 than for GPTanam, especially with the classes MVA and Sport Accident, where the average gain of the micro F1-score per class was 0.07, as shown in Figure 2. Performance for the class “Accident of exposure to bodily fluids” did not improve for TF-IDF/SVM, CamemBERT, and FlauBERT when complex and ambiguous content was removed from the test data set. The performance of GPTanam did not improve for the classes “Foreign body on the eye” and “Other trauma,” but the F1-scores were already very high for these classes—0.97 and 0.98, respectively. Performance was slightly improved for “Assault,” “Fall,” “MVA,” “Sport Accident,” and “Other trauma” when potentially complex and ambiguous content was removed from the test data set for all the models as seen in Multimedia Appendix 5 and the confusion matrix in Multimedia Appendix 6.

**Error analysis results.**

**Accident of exposure to bodily fluids**

The bigram analysis showed that the keywords “contact blood” were absent in the top 10 bigrams in the incorrectly classified clinical notes, whereas the unigrams analysis showed that “HIV” is the ninth unigram (after “aes,” “blood,” “needle,” “source,” “intercourse,” “dakin,” “work,” and “sexual”).

**Assault**

Regarding the class “Assault,” the top 3 bigrams were “physical assault,” “declare having,” and “punch” (*coup poing* in French) for the correctly classified clinical notes, whereas “left hand,” “hand trauma,” and “mechanical fall” were the most frequent bigrams. The verification of the 18 clinical notes manually annotated as “Assault” showed that for 11 (61%) of them, the label predicted by the model was correct (n=1, 9% fall; n=8, 73% self-harms; n=1, 9% motor vehicle accident [MVA]; and n=1, 9% sport accident paintball).

**MVA**

The acronym “mva” (n=700, 26%) was the most represented unigram in the correctly classified corpus, whereas “pain” was the most represented unigram in the clinical notes classified as not MVA. When analyzing the 6 incorrectly classified clinical notes, 3 (50%) of them were wrongly labeled as they were in fact referring to an assault, a fall, and a basketball accident. The 3 (50%) remaining clinical cases contained 2 types of traumas such as falling on the street.

**Foreign body in the eye**

The unigram analysis for this class showed that the unigrams “eye” and “the eye” were the most represented (n=140, 13%), whereas “left” and “hear” were the top 2 unigrams in the clinical notes classified as not being “foreign body in the eye.” In fact, one of these clinical notes was related to a foreign body in the heart, and 2 others were assault without mention of eye trauma.

**Fall**

The top 3 bigrams for the correctly classified clinical notes were “mechanical fall,” “loss of consciousness,” and “cranial trauma” and “right ankle,” “ankle trauma,” “left ankle” for the incorrectly classified ones. In total, 21 of the incorrectly classified clinical notes encompassed a double mechanism of trauma: 1 (5%) sport accident, 16 (76%) MVAs, and 4 (19%) assaults involving a fall were present. A total of 9 notes mentioned back pain, ankle and knee twists, pain while getting off of a truck, or a patient found at the bottom of the stairs without mention of falling.

**Intentional injury**

The most frequent unigrams and bigrams were different between the correctly and incorrectly classified clinical notes. The most represented unigrams and bigrams were, respectively, “imv” (“voluntary drug intoxication” in French) and “suicide attempt” in the correctly classified corpus of clinical notes, whereas “hand” and “punch given” were the most common in the incorrectly classified notes. Indeed, the model classified 10 clinical notes as assault, whereas these clinical notes were related to a patient having punched something or himself.

**Sport**

In the correctly classified clinical notes, the most frequent unigrams were “pain,” “left,” and “trauma” and the most frequent bigrams were “right ankle,” “functional impotence,” and “left knee.” In the incorrectly classified notes, the most frequent unigrams and bigrams were, respectively, “fall,” “trauma,” and “bike” and “bike fall,” “right knee,” and “knee pain.” A total of 13 falls occurred while biking (the notes did not mention the place) and were classified as MVA. Five incorrectly classified notes were eye trauma while practicing sports.

Removing complex and ambiguous clinical notes were associated with an increase of performance for all the models; the average gain of *F*_1_-scores was 0.04 for TF-IDF/SVM, CamemBERT, FlauBERT, and BelGPT2. The average gain of the micro *F*_1_-score was 0.01 for GPTanam, which seems to be more robust in classifying complex and ambiguous content.

The difference in performance when potentially complex and ambiguous content was considered was greater for TF-IDF/SVM, CamemBERT, FlauBERT, and BelGPT2 than for GPTanam, especially with the classes MVA and Sport Accident, where the average gain of the micro *F*_1_-score per class was 0.07, as shown in Figure 2. Performance for the class “Accident of exposure to bodily fluids” did not improve for TF-IDF/SVM, CamemBERT, and FlauBERT when complex and ambiguous content was removed from the test data set. The performance of GPTanam did not improve for the classes “Foreign body on the eye” and “Other trauma,” but the *F*_1_-scores were already very high for these classes—0.97 and 0.98, respectively. Performance was slightly improved for “Assault,” “Fall,” “MVA,” “Sport Accident,” and “Other trauma” when potentially complex and ambiguous content was removed from the test data set for all the models as seen in Multimedia Appendix 5 and the confusion matrix in Multimedia Appendix 6.

## Discussion

### Transformers: A New State of the Art

The transformers showed interesting results when applied to free-text data from the ED of the Bordeaux University Hospital; a GPT-2 model with a French tokenizer and a self-supervised training step on a domain-specific corpus in addition to a large French corpus reached an average micro *F*_1_-score of 0.969. This model showed better performance than TF-IDF/SVM and the other transformer models on average metrics and for all classes. In 2018, when reviewing deep learning algorithms for clinical natural language processing, the study by Wu et al projected the rise in the popularity of transformer models [36]. However, some studies showed that traditional approaches, when tailored to the specific language and structure of the text inherent to the classification task, can achieve or exceed the performance of more recent ones based on contextual embeddings such as BERT [37]. Further study could involve comparing our model’s performance with that of bidirectional long short-term memory with pretrained embeddings such as Word2Vec or transformer embeddings and CNN.

### Self-supervised Training on Domain-Specific Corpus and Tokenizer

The decision to use pretrained models on French corpora with a French tokenizer has probably contributed to the global performance of the chosen transformer models. General language transformer models pretrained on a cross-domain text corpus in a given language have recently flourished. BelGPT2 was the first GPT-2 model fine-tuned on a French heterogeneous corpus (CommonCrawl, French Wikipedia, and EuroParl) released on the Hugging Face platform. The self-supervised training of transformers in a specific domain can improve the performance of tasks such as classification [38], text generation [39], and predicting hospital readmission [40]. Despite many experiments using BERT, GPT-2 has not been studied as extensively as BERT yet. Our team showed that the amount of data required to achieve a given level of performance (area under the curve >0.95) was reduced by a factor of 10 when applying self-supervised training on emergency clinical notes to a binary classification task [41]. Here, we confirmed the benefits of a self-supervised training step on a domain-specific corpus. However, it is questionable whether this approach will be applicable when extending the TARPON project to data from other EDs in France, as each region or ED uses a specific language in addition to the medical language, which uses many abbreviations that can vary locally (eg, assault is written as “brawl” in Bordeaux and “hep” means hepatitis). A possible solution would be to train the model on a corpus resulting from the extraction of ED notes at a national level. Similarly, the treatment of medical concepts and abbreviations remains an area for improvement, as not all EDs use the same abbreviations in the same context. The use of ontologies developed in the field of emergencies could constitute an area for improvement. Transformers have also recently been tested for the identification and replacement of abbreviations, with good results for BERT [42,43]; however, there has not yet been a test on data from a mixture of common language and medical terms in French.

In addition, because the authors who proposed the CamemBERT model did not compare the performance of different models from the OSCAR, CCNET, and Wikipedia data sets in a classification task, a future study could compare the different sets in our database in this regard. While we have only used the basic models of CamemBERT, FlauBERT, and GPT-2, it would be appropriate to test the different sizes of pretraining data sets on a classification task as well as the different sizes of models. Indeed, Martin’s [44] team has shown that the standard CamemBERT model (110 million parameters) trained on all 138 GB of OSCAR text does not massively outperform the model trained “only” on the 4 GB sample in morphosyntactic labeling, syntactic parsing, named entity recognition, and natural language inference. One perspective considered is to test different models of French transformers that have been released since CamemBERT, FlauBERT, and BelGPT2 such as Pagnol and BARThez.

### Taxonomy

The performance of the models improved when we excluded the clinical notes that we considered the most complex and ambiguous from our test data set. The classification error analysis showed that when clinical notes encompassing 2 mechanisms of trauma (ie, “fall from bike on the street”) were removed from the test data set, the models performed better. This expected result shows that since the advent of transformers, the margin of progress in a free-text classification task is nowadays low. This behavior was less important with GPTanam, which seems to have benefited from the self-supervised pretraining phase for reducing classification errors by learning semantic representations beforehand. However, the annotation grid created for the project is partly responsible for some classification errors in the sense that there are areas of semantic overlap between classes. In addition, the coding system used did not allow for the coding of several traumatic mechanisms (eg, a collision between 2 individuals followed by a fall). To be able to account for these situations, a new coding system will be used for the next phases of the project, using the recently released version of trauma classification grid used by the FEDORU (Fédération des Observatoires Régionaux des Urgences) and OSCOUR.

### Improving Trauma Public Health Surveillance

The costs of injury and morbidity are immense not only in terms of lost economic opportunities and demands on national health budgets but also in terms of personal suffering [45]. However, few countries have surveillance systems that generate reliable information on the nature and extent of injuries, especially nonfatal injuries. The traditional view of injuries as “accidents” or random events has resulted in the historical neglect of this area of public health [46]. However, in recent decades, public health officials have been recognizing traumas as preventable events and have been promoting evidence-based interventions for the prevention of traumas worldwide [47]. Many injury interventions are already in place (eg, transportation requirements such as setting speed limits, safe automobile design, seatbelt and other safety restraint use, and use of helmet and other protective equipment) and have achieved significant public health improvements, including the reduction of trauma occurrence [48].

The automatic labeling of ED clinical notes will contribute to an effective real-time public health surveillance system for traumas. Future steps encompass deployment in hospitals’ IT departments in Gironde, France, at first, and then at a national scale.

### Conclusions

Transformers have shown great effectiveness in a multiclass classification task on complex data encompassing narrative, medical data, and jargon. The choice of this type of architecture in the automatic processing of ED summaries to create a national observatory is relevant. Applying a self-supervised training step on a specific domain corpus has substantially improved the classification performance of a French GPT-2 model. The next labeling strategy within the framework of the TARPON project will be carried out using a standardized trauma classification tool, which will allow a more precise classification of trauma mechanisms owing to a clearer delineation between the different classes (little overlap of semantic fields). The objective is eventually to have a single code for ED summaries, including several variables (eg, place of occurrence, activity during the trauma, and role in a road accident). It is necessary to investigate the possibility of making predictions with a model trained on each variable or using a single model trained on all variables. If the latter method is chosen, a larger model of GPT-2 will probably be required. Furthermore, the expansion of acronyms is under consideration in the automation pipeline.

## Appendix

Multimedia Appendix 1 Composite variable creation. MVA: motor vehicle accident.

Multimedia Appendix 2 Emergency department electronic health record visualization with clinical note translated in English.

Multimedia Appendix 3 Example of 2 clinical notes artificially generated by GPTanam right after the self-supervised training step with a setup of maximum 40 tokens generated. Clinical notes in French are on the left, and translated notes in English are on the right.

Multimedia Appendix 4 Confusion matrix for the GPTanam model on the complete test data set. Ratio and percentage of correctly classified clinical notes per class are given. MVA: motor vehicle accident.

Multimedia Appendix 5 Average macro-precision and micro F1-score for each model for the test data set without complex/ambiguous content in clinical notes. MVA: motor vehicle accident; SVM: support vector machine; TD-IDF: term frequency–inverse document frequency;.

Multimedia Appendix 6 Confusion matrix for the GPTanam model on the test data set without complex/ambiguous content in clinical notes. Ratio and percentage of correctly classified clinical notes per class are given. MVA: motor vehicle accident.

## Abbreviations

BERT: Bidirectional Encoder Representations Transformer
CNN: convolutional neural network
ED: emergency department
EHR: electronic health record
FEDORU: Fédération des Observatoires Régionaux des Urgences
GPT-2: Generative Pretrained Transformer-2
ICD: International Classification of Diseases
MVA: motor vehicle accident
SurSaUD: Surveillance Sanitaire des Urgences et des Décès
SVM: support vector machine
TARPON: Traitement Automatique des Résumés de Passage aux urgences dans le but de créer un Observatoire National
TF-IDF: term frequency–inverse document frequency

## Data Availability

The data set is not available because of patient privacy restrictions. However, the model may be shared with qualified researchers from academic or university institutions upon request via the corresponding author.
