# Supplementary figures and images for: Deep Learning Transformer Models for Building a Comprehensive and Real-time Trauma Observatory: Development and Validation Study
Source: JMIR AI. 2023 Jan 12;2:e40843. doi: 10.2196/40843 (PMC11041521; doi:10.2196/40843)

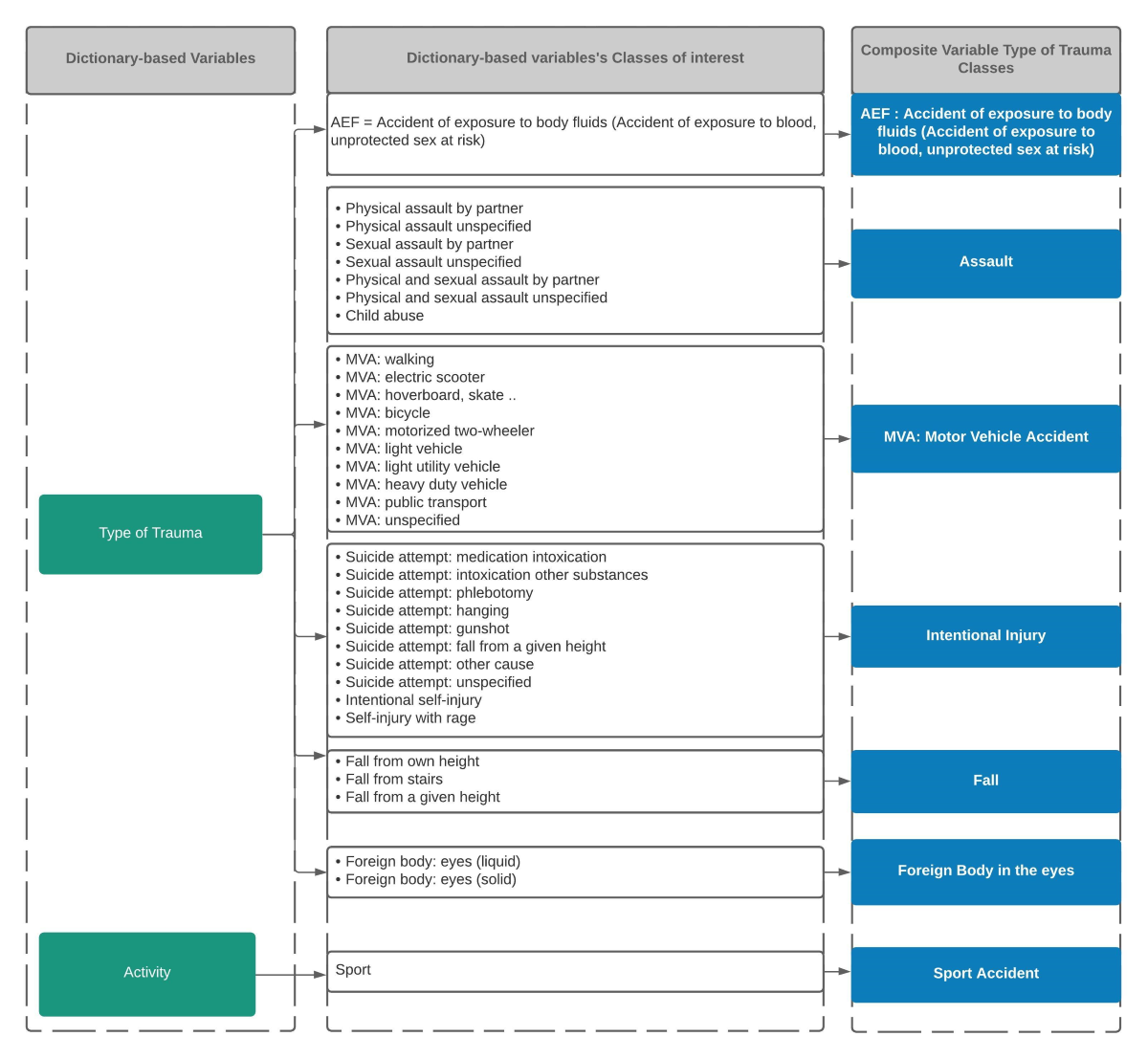

Supplement: Multimedia Appendix 1 [file ai_v2i1e40843_app1.png]

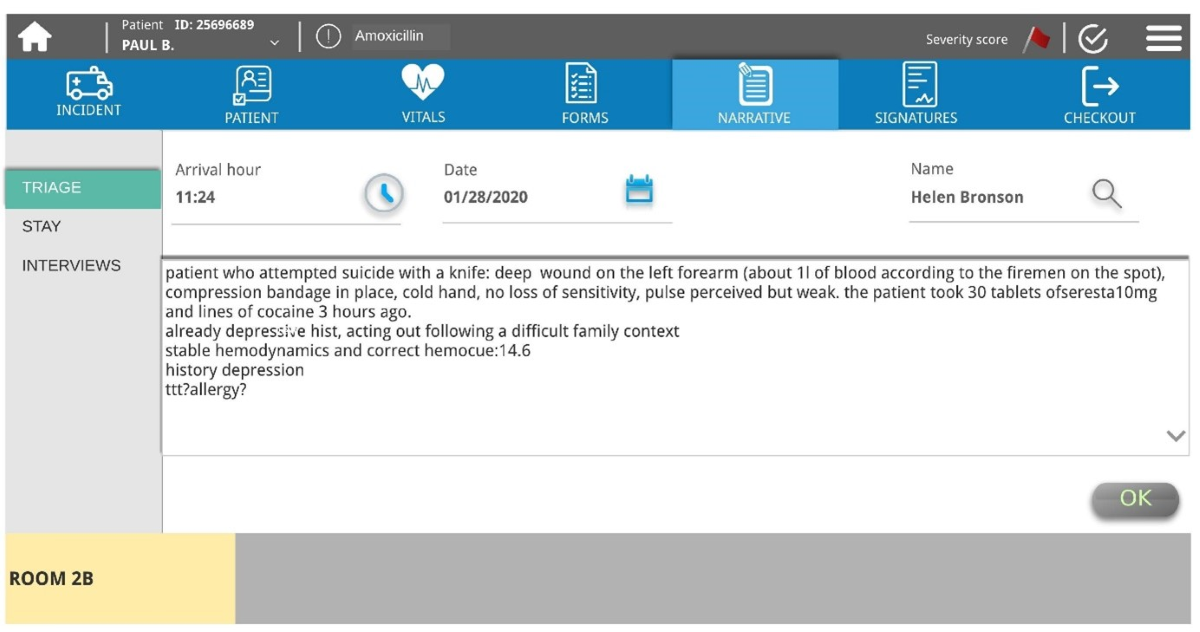

Supplement: Multimedia Appendix 2 [file ai_v2i1e40843_app2.png]

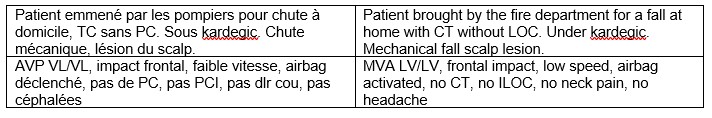

Supplement: Multimedia Appendix 3 [file ai_v2i1e40843_app3.png]

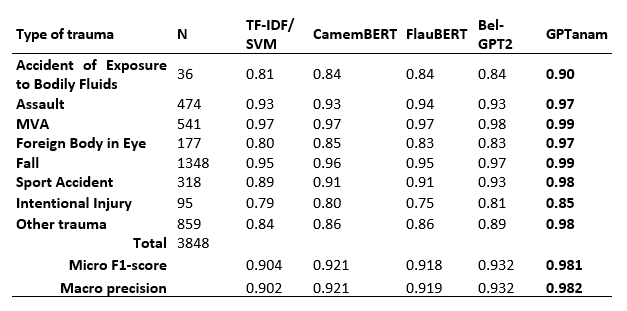

Supplement: Multimedia Appendix 5 [file ai_v2i1e40843_app5.png]
